# Geriatric Nutritional Risk Index is related to the risk of stroke‐associated pneumonia

**DOI:** 10.1002/brb3.2718

**Published:** 2022-07-18

**Authors:** Caijun Dai, Dan Yan, Minjie Xu, Qiqi Huang, Wenwei Ren

**Affiliations:** ^1^ Department of Pulmonary and Critical Care Medicine, Affiliated Jinhua Hospital, Zhejiang University School of Medicine Jinhua Municipal Central Hospital Jinhua China; ^2^ Department of Neurology The First Affiliated Hospital of Wenzhou Medical University Wenzhou China; ^3^ Department of Cardiac Care Unit The First Affiliated Hospital of Wenzhou Medical University Wenzhou China

**Keywords:** Geriatric Nutritional Risk Index (GNRI), pneumonia, stroke

## Abstract

**Background:**

Stroke‐associated pneumonia (SAP) occurs frequently after a stroke. Geriatric Nutritional Risk Index (GNRI) is a valuable indicator of elderly individuals’ nutritional status. This research was designed to obtain insight into the link between GNRI and SAP.

**Methods:**

Patients with acute ischemic stroke (AIS) were categorized into the SAP and non‐SAP groups. GNRI scores were divided into four layers: Q1, GNRI < 82; Q2, 82≤ GNRI < 92; Q3, 92≤ GNRI ≤98; Q4, GNRI > 98. To identify the independent risk and protective factors of developing SAP, logistic regression analyses were conducted. Additionally, we utilized the restricted cubic spline (RCS) analysis to test the effect of GNRI on the SAP risk.

**Results:**

The SAP group showed lower GNRI scores than the non‐SAP group (96.88 ± 9.36 vs. 100.88 ± 8.25, *p*  <  0.001). According to the logistic regression model, the Q1 and Q2 layers showed a higher risk of SAP than the Q3 layer, while the Q4 layer showed a lower SAP risk (all *p* < 0.05). Besides, the RCS model found that the risk of SAP dropped dramatically as GNRI scores increased, which got stable when the GNRI score was more significant than 100.

**Conclusion:**

Lower GNRI scores were linked to a higher prevalence of SAP. In clinical practice, GNRI showed predictive value for SAP, which could be helpful in early SAP intervention and therapy.

## Graphical abstract

Our study demonstrated that GNRI scores at admission were associated with the risk of SAP, and lower GNRI score indicated a higher risk of SAP. Besides, there existed a non‐linear relationship between GNRI and the risk of SAP. GNRI showed predictive value for SAP, which could be useful for early SAP intervention and therapy.

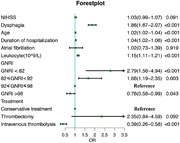

## 1 INTRODUCTION

Acute ischemic stroke (AIS) is considered to be a major cause of mortality and permanent disability in the world (Benjamin et al., 2019). Case fatality and disability rates of AIS for one year were 6.0% and 14.2%, respectively, in a nationwide hospital‐based cohort study in China (Tu et al., 2021). Stroke‐associated pneumonia is one of the most prevalent complications in AIS patients (Hilker et al., 2003), which may lead to increased mortality, prolonged hospitalization, poor long‐term clinical outcomes, and increased economic burden (Ali et al., 2018; Hilker et al., 2003; Katzan et al., 2003; Teh et al., 2018; Verma, 2019). Therefore, SAP has attracted growing attention in recent years. The occurrence of SAP is multifactorial (Al‐Khaled, 2019). A better understanding of the influencing factors of SAP will be vital for driving the rate of pneumonia in AIS patients down.

GNRI is a new, easy‐available and effective nutritional evaluation index for nutritional risk assessment (Bouillanne et al., 2005). This index can predict the complications induced by malnutrition as well (Cereda et al., 2009). Predictions of some diseases incidence and prognosis have been made through GNRI in earlier investigations, including postoperative survival rate of esophageal cancer (Kubo et al., 2019), postoperative complications of gastric cancer (Kushiyama et al., 2018), postoperative bleeding of the pancreas (Funamizu et al., 2020), mortality of heart failure (Li et al., 2021), the prognosis of coronavirus disease(COVID‐19) (Lidoriki et al., 2020; Recinella et al., 2020), and poststroke cognitive impairment (Lee et al., 2021). Besides, studies have also found that malnutrition can lead to complications such as decreased immunity and infection (Keusch, 2003). For example, previous studies had found that malnutrition was a risk factor for community‐acquired pneumonia (Almirall et al., 2017), and could affect the clinical prognosis of community‐acquired pneumonia (Wei et al., 2020).

Malnutrition is widespread in patients with stroke (Henke et al., 2017). Previous research on AIS patients indicated that malnutrition was a significant predictor for poor clinical outcomes (Yoo et al., Jan, 2008). Moreover, another earlier study had found that GNRI was strongly related to prolonged hospitalization and poor prognosis after stroke (Kang et al., 2020). However, the relationship between GNRI and SAP has not been investigated yet. Therefore, this study aimed to explore whether GNRI played a role in SAP.

## 2 METHODS

### 2.1 Study population

**FIGURE 1:**
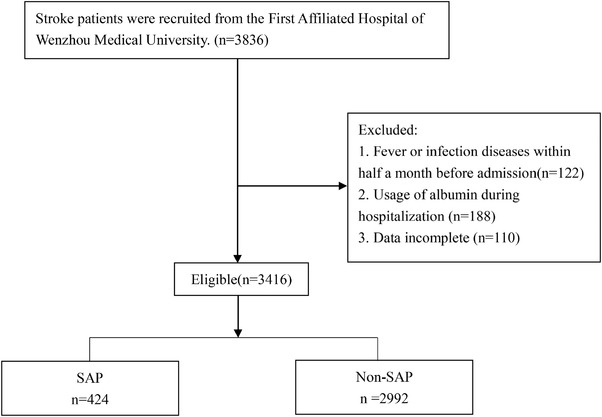
Flowchart of participants. SAP: stroke‐associated pneumonia

In total, 3836 AIS patients were screened from the First Affiliated Hospital of Wenzhou Medical University between February 2013 and January 2021. Finally, 3416 patients were recruited and assigned to two groups: the SAP group (*n* = 424) and the non‐SAP group (*n* = 2992) (Figure 1). The criteria for inclusion include the following: (1) diagnosis of AIS confirmed by magnetic resonance imaging; (2) hospitalization within 1 week of symptom onset; and (3) age ≥18. The criteria for exclusion include the following: (1) suffered from fever or infection diseases within half a month before admission; (2) usage of albumin during hospitalization; and (3) data incomplete.

The institutional review board of First Affiliated Hospital of Wenzhou Medical University (KY2021‐R077) had approved this study. Because all data were collected retrospectively and anonymized, informed consent could be waived, which had been approved by Ethics Committee.

### 2.2 Data collection

We obtained the general information of these patients from the hospital registry systems, such as age, gender, body mass index (BMI), history of atrial fibrillation, hypertension, diabetes mellitus, smoking, and drinking. Furthermore, we collected the clinical features, including laboratory findings on admission (albumin, leukocyte, estimated glomerular filtration rate [eGFR]), stroke classification (TOAST criteria), length of hospital admissions, the severity of dysphagia, treatment method, and neurologic impairment assessed by the National Institutes of Health Stroke Scale score (NIHSS). Stroke‐associated pneumonia (SAP) was diagnosed by two experienced neurologists during the first seven days of hospitalization after stroke onset, according to the modified Centers for Disease Control and Prevention criteria of hospital‐acquired pneumonia, confirmed by the radiological imaging, clinical signs, and laboratory measures of lung infection (Garner et al., 1988).

The severity of dysphagia and neurologic impairment were assessed within 24 h of admission. The diet (general diet, viscous paste meal, semiliquid diet, nasogastric tube feeding) was determined based on the results of the dysphagia assessment. Fasting blood samples were obtained on the second morning after admission to measure biochemical indicators.

GNRI is a simple and effective risk index to evaluate patients’ nutritional status (Bouillanne et al., 2005). The GNRI was computed by the following equation: 1.489 × albumin (g/L) + 41.7 × present weight/ideal weight(kg). Ideal weight was computed by the Lorentz equation (men: Height – 100 – [(Height – 150)/4]; women: Height – 100 – [(Height – 150)/2.5]). According to GNRI scores, GNRI was divided into four nutrition‐related risk layers (Q1–Q4): Q1, a major‐risk layer with GNRI < 82; Q2, a moderate‐risk layer with 82≤ GNRI < 92; Q3, a low‐risk layer with 92≤ GNRI ≤98; and Q4, no‐risk layer with GNRI > 98 (Bouillanne et al., 2005; Cereda et al., 2006).

### 2.3 Statistical analyses

To summarize baseline characteristics, descriptive statistics were used. For group comparisons, the *t*‐test, χ^2^ test, or Fisher's exact probability method were utilized. The mean and standard deviation are used to express continuous variables. Categorical variables are expressed in terms of the number of cases and percentages. The independent determinants for SAP were determined using multivariable logistic regression analysis, and the findings were presented as a forest plot. A restricted cubic spline (RCS) was employed to clarify the link between GNRI and the SAP risk. R v3.5.1 was used to conduct all statistical analyses. Statistical significance was defined as *p* < 0.05.

## 3 RESULTS

### 3.1 Characteristics between SAP and non‐SAP groups

As shown in Table 1, 3416 eligible AIS patients were enrolled in this study, including 424 (12.4%) patients with SAP and 2992 (87.6%) patients without SAP. Compared with the non‐SAP group, patients in the SAP group were older, with lower GNRI scores on admission, more extended hospitalization, higher incidence of atrial fibrillation, higher NIHSS scores, lower BMI, more severe dysphagia and a lower percentage of intravenous thrombolysis treatment (all *p* < 0.05). Furthermore, leukocyte counts were significantly more significant in the SAP group, whereas albumin levels were lower (all *p* < 0.05).

**TABLE 1:** Comparisons of clinical characteristics

Variables	Non‐SAP (*n* = 2992)	SAP (*n* = 424)	*p*
**Demographic characteristic**			0.36
Gender, *n* (%)			
Male	1925 (64.34)	283 (66.75)	
Female	1067 (35.66)	141 (33.25)	
Age	65.89 ± 11.46	71.83 ± 11.04	<0.001
BMI	23.96 ± 3.29	23.31 ± 3.70	<0.001
Duration of hospitalization	10.31 ± 4.95	13.24 ± 6.92	<0.001
Hypertension, *n* (%)	2095 (70.02)	299 (70.52)	0.878
Diabetes, *n* (%)	907 (30.31)	138 (32.55)	0.38
Atrial fibrillation, *n* (%)	315 (10.53)	81 (19.1)	<0.001
Drinking history, *n* (%)	1001 (33.46)	139 (32.78)	0.826
Smoking history, *n* (%)	1216 (40.64)	161 (37.97)	0.319
GNRI	100.88 ± 8.25	96.88 ± 9.36	<0.001
GNRI, *n* (%)			<0.001
92 ≤ GNRI ≤ 98	798 (26.67)	118 (27.83)	
GNRI < 82	44(1.47)	30 (7.08)	
82 ≤ GNRI < 92	296 (9.89)	90 (21.23)	
GNRI > 98	1854(61.97)	186 (43.87)	
Dysphagia, *n* (%)			<0.001
Ordinary diet	1827 (61.06)	115 (27.12)	
Viscous paste meal	854 (28.54)	108 (25.47)	
Semiliquid diet	84 (2.81)	17 (4.01)	
Nasal feeding	227 (7.59)	184 (43.4)	
NIHSS, median (Q1, Q3)	2 (1, 4)	3 (1, 7)	<0.001
TOAST, *n* (%)			0.813
Large artery atherosclerosis	1051 (35.13)	152 (35.85)	
Other	1941 (64.87)	272 (64.15)	
Treatment			<0.001
Endovascular thrombectomy	12 (0.40)	8 (1.90)	
Intravenous thrombolysis	413 (13.80)	39 (9.20)	
Conservative treatment	2567 (85.80)	377 (88.90)	
**Laboratory parameters**			
Leukocyte (10^9^/L)	7.13 ± 2.06	8.39 ± 2.70	<0.001
Albumin (g/L)	37.58 ± 3.09	35.78 ± 3.84	<0.001
eGFR	76.16 ± 28.89	88.17 ± 82.07	0.106

BMI: body mass index; SAP: stroke‐associated pneumonia; GNRI: Geriatric Nutritional Risk Index; NIHSS: National Institutes of Health Stroke Scale score; eGFR: estimated glomerular filtration rate.

### 3.2 GNRI and SAP

**FIGURE 2:**
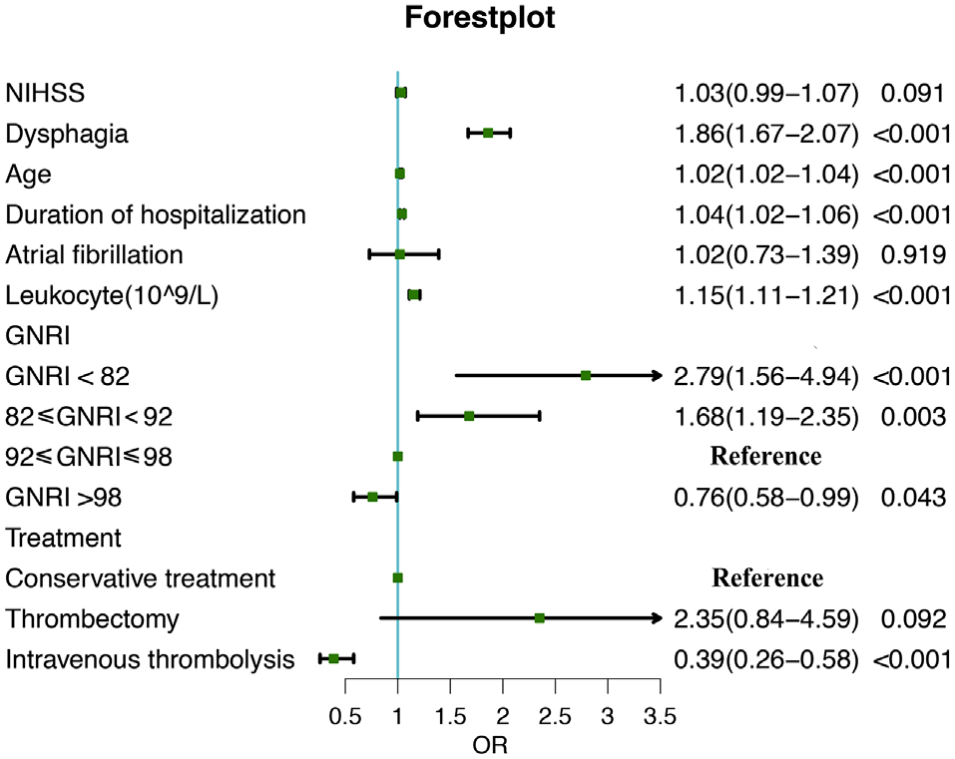
Forest plot of odds ratios (OR) for SAP. An OR > 1 meant an increased risk of SAP, and an OR < 1 meant the opposite. SAP: stroke‐associated pneumonia; GNRI: Geriatric Nutritional Risk Index; NIHSS: National Institutes of Health Stroke Scale score

Multivariable logistic regression analysis revealed that patients in Q1 (OR: 4.61; 95% CI: 2.77−7.59, *p* < 0.001) and Q2 (OR: 2.06; 95% CI: 1.51−2.79, *p* < 0.001) GNRI layers were more susceptible to develop SAP compared with Q3 GNRI layer. Moreover, the Q4 GNRI layer showed a decreased risk of SAP compared with the Q3 GNRI layer (OR: 0.67; 95% CI: 0.53−0.87, *p* < 0.05). These findings remained unchanged after adjusting for the potential confounders (age, atrial fibrillation, NIHSS scores, length of hospitalization, leukocyte, dysphagia, and treatment method) (Figure 2).

When GNRI score was included as a continuity variable in the model, the findings remained unchanged in both unadjusted (OR: 0.94, 95% CI: 0.93–0.96, *p* < 0.001) and adjusted model (OR: 0.96, 95% CI: 0.95–0.97, *p* < 0.05). In our study, 452 patients received intravenous thrombolysis treatment, 20 patients received endovascular thrombectomy, and 2944 received conservative treatment. The GNRI scores of the three groups were 100.49 ± 9.20, 97.65 ± 9.13, and 100.38 ± 8.39, respectively, and there is no difference among these groups (*p* > 0.05). Furthermore, 13 patients were successfully reperfused after thrombectomy, and 7 were not successfully reperfused. The median GNRI scores of patients with successfully reperfused and not successfully reperfused were 98 (IQR 90–106.5) and 97 (IQR, 93–103), respectively, and no difference was found (*p* > 0.05).

### 3.3 The nonlinear link between GNRI and SAP risk

**FIGURE 3:**
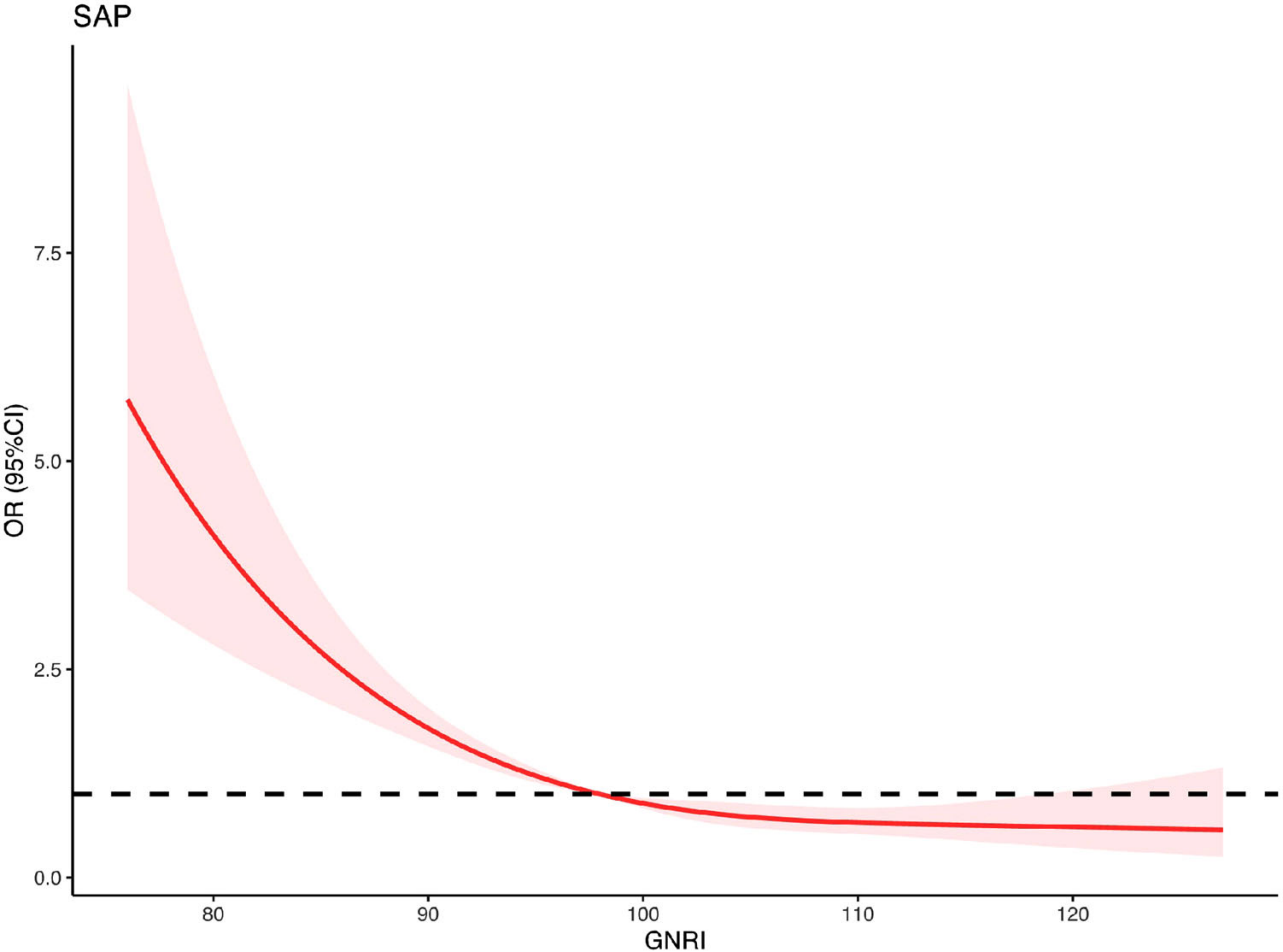
Association between GNRI and risk of SAP using restricted cubic spline (RCS) analysis. SAP: stroke‐associated pneumonia; GNRI: Geriatric Nutritional Risk Index

The RCS model revealed a nonlinear link between GNRI and the SAP risk (*p* for nonlinearity < 0.001, Figure 3). The RCS model showed that the risk of SAP dropped dramatically as GNRI scores increased, which got stable when the GNRI score was greater than 100.

## 4 DISCUSSION

To our knowledge, this is the first study to examine the link between GNRI and SAP. The major findings are as follows: (1) a lower GNRI score indicated a higher SAP risk and (2) there existed a nonlinear relationship between GNRI and SAP risk.

Previous studies have found that stroke patients often suffer from malnutrition, and the patients’ nutritional status in the acute phase may affect the recovery of physical functional (Irisawa & Mizushima, 2020; Jensen et al., 2010). Several nutrition risks screening tools, including the Simplified Nutritional Appetite Questionnaire (SNAQ), and the Malnutrition Universal Screening Tool (MUST), have been developed to assess nutritional statuses throughout many hospitals. However, these tools need not only the patients’ cooperation but also their recent weight loss (Dent et al., 2019). In addition, these nutritional assessment methods are subjective and arbitrary, requiring well‐trained professionals of health care or patients with normal cognitive function. Because some AIS patients may be unconscious, these tools may not suitable for screening all patients of AIS. GNRI is an objective and simple assessment indicator for screening nutritional status. GNRI scores can be easily and automatically calculated in the electronic medical record system. By comparing various nutrition‐related parameters, Abd‐El‐Gawad et al. (2014) found that GNRI exhibited great predictive ability in evaluating nutrition and predicting the prognosis of nutrition‐related complications.

Accumulating studies have observed that malnutrition could increase the risk of infection (Felblinger, 2003; Keusch, 2003). For example, several studies indicated that albumin was a significant risk factor for hospital‐acquired pneumonia (Dziedzic et al., 2006; Yang et al., 2020). Besides, another study found that GNRI was a risk factor for surgical site infection after pancreaticoduodenectomy (Funamizu et al., 2020). In the present study, GNRI was an independent risk factor for SAP in AIS patients. SAP was more likely to occur in patients with a low GNRI score (<92), while those with high GNRI scores (≥98) were less prone to SAP. Possible explanations are as follows. GNRI is computed by weight, height, and serum albumin. Serum albumin, an indirect indicator of inflammation, is usually used to evaluate nutrition status (Eckart et al., 2020). Serum albumin level at admission is significantly negatively correlated with infectious complications of stroke patients (Gariballa et al., 1998). In addition, albumin has the ability to stimulate the response of immune cells (Gioannini et al., 2002). Hypoalbuminemia may aggravate the immunosuppressive status due to the failure to activate the immune response, further increasing the risk of pneumonia (Arroyo et al., 2014). Moreover, albumin has been considered to have antioxidant activity and anti‐inflammatory properties (Arroyo et al., 2014; Idicula et al., 2009; Kawai et al., 2018), which may reduce the risk of infection. It is well known that albumin is used for nutritional assessment and prediction of clinical outcomes after acute stroke (Gariballa et al., 1998). However, serum albumin has a limited role in detecting sharp dietary changes because of its long half‐life (Lipkin & Bell, 1993). Moreover, serum albumin concentration negatively correlates the volume of extracellular fluid. Meanwhile, body weight can also be affected by the hydration status (Jones et al., 1998). Therefore, the combination of serum albumin and body weight in GNRI may minimize the effects of confounding factors such as hydration status, which could better predict the adverse prognosis related to nutrition. Multiple biomarkers were associated with inflammation and showed the ability to predict outcomes or SAP events in stroke, such as CRP and IL‐6 (Lu et al., 2015; Yang et al., 2020). A study indicated that IL‐6 was related to SAP via activating the production of acute‐phase proteins and CRP in AIS (Yang et al., 2020). Besides, acute stroke may induce depression of the immune system with an elevated susceptibility to infections (Haeusler et al., 2008), which may be partly promoted by increased IL‐6, for the reason that IL‐6 plays a role in determining cellular immunity (Yang et al., 2020). Furthermore, it was previously demonstrated that IL‐6 and TNF‐a were also associated with the outcomes of SAP (Yu et al., 2017). Unlike the inflammation biomarkers, such as IL‐6 or CRP, GNRI was a nutrition‐associated biomarker. A previous study found that malnutrition can lead to a series of complications such as decreased immunity and infection (Keusch, 2003). Similar to previous studies, our study first revealed that GNRI was associated with SAP. Malnutrition is one of the most common causes of immune system deterioration (Bourke et al., 2016). Besides, immunosuppression after stroke may lead to the downregulation of the systemic cellular immune response and the rapid reduction of lymphocyte subsets, monocytes, pulmonary macrophages, and epithelial cells (Chamorro et al., 2012; Dirnagl et al., 2007; Engel et al., 2015; Prass et al., 2003), thereby contributing to the increased susceptibility to SAP (Hoffmann et al., 2017).

In previous studies, malnutrition has been identified as a risk factor for community‐acquired pneumonia (Almirall et al., 2017). GNRI could affect the clinical prognosis of community‐acquired pneumonia (Wei et al., 2020). Similar to previous studies, our study revealed that a lower GNRI score indicated a higher SAP risk. Moreover, both malnutrition and GNRI were predictors of poor clinical outcomes in AIS patients (Kang et al., 2020). Unfortunately, this study had not evaluated the relationship between GNRI and clinical outcomes. However, our study had firstly presented that GNRI was associated with SAP, and previous studies had revealed that SAP was associated with increased mortality and poor clinical outcomes in stroke patients (Hilker et al., 2003; Katzan et al., 2003; Teh et al., 2018). We supposed that GNRI may also be associated with the outcomes of stroke patients. Further research is needed to confirm this hypothesis. Given these observations, we also supposed that malnutrition may lead to SAP, which may subsequently lead to poor outcomes of stroke. Therefore, GNRI may help the physician identify the risk of SAP, and intervene timely, which may reduce the incidence of SAP, and ultimately improve the outcomes of stroke patients. A previous nationwide survey in China showed that the case fatality and disability rates of AIS were lower than before, and stroke outcomes appeared to have improved in these years (Tu et al., 2021). On the one hand, it may be owing to the better treatments after stroke. On the other hand, improvement in stroke patients’ nutritional status may also contribute to better outcomes. Therefore, it would be very interesting to see whether the GNRI also played a role in the outcomes of stroke. We will further explore this issue in the future.

There are several limitations to this research that deserve attention. To begin, since this is a cross‐sectional study, we were unable to conclude a causal relationship between GNRI and SAP. Secondly, we could not identify the fluctuation of GNRI throughout hospitalization for it was only detected once at admission. Third, we did not collect the data on mortality and prognostic outcomes, so we cannot assess the association between the Geriatric Nutritional Risk Index (GNRI), SAP, and stroke outcome or mortality. Future studies were needed to elucidate those relations. Fourth, inflammatory biomarkers, such as IL‐6 or CRP, were not collected in this study. Therefore, GNRI cannot be compared to other inflammatory biomarkers in this study. And we were unable to evaluate the effect of intravenous thrombolysis or endovascular thrombectomy on these biomarkers. Further studies on the connection between GNRI and inflammatory biomarkers were needed in the future, which may help us better understand the role of GNRI in stroke. Lastly, the patients in this study were recruited from a center in China. Therefore, the findings of this study may not apply to all populations, and external validation is necessary to confirm our results in the future.

## 5 CONCLUSION

In conclusion, this study indicates that low GNRI is an essential and independent risk factor for SAP in AIS patients. It may provide important predictive value for clinicians to better evaluate the risk of developing pneumonia in stroke patients, and intervene as soon as possible to improve the outcomes of stroke patients.

### PEER REVIEW

The peer review history for this article is available at https://publons.com/publon/10.1002/brb3.2718

## CONFLICT OF INTEREST

The authors have no conflicts of interest to disclose.

## AUTHOR CONTRIBUTIONS

WR and QH contributed to the conception and design of the study. CD and DY collected the clinical data. WR and QH contributed significantly to manuscript preparation. CD, DY, and MX wrote the manuscript and performed the analysis with constructive discussions.

## Data Availability

The data analyzed during the study are available from the corresponding author on reasonable request.
